# Serum Calponin 3 Levels in Patients with Systemic Sclerosis: Possible Association with Skin Sclerosis and Arthralgia

**DOI:** 10.3390/jcm10020280

**Published:** 2021-01-14

**Authors:** Hirohito Kotani, Ayumi Yoshizaki, Kazuki M. Matsuda, Yuta Norimatsu, Ai Kuzumi, Maiko Fukayama, Takemichi Fukasawa, Satoshi Ebata, Asako Yoshizaki-Ogawa, Yoshihide Asano, Koji Oba, Shinichi Sato

**Affiliations:** 1 Department of Dermatology, The University of Tokyo Graduate School of Medicine, Tokyo 113-8655, Japan; kotanih-der@h.u-tokyo.ac.jp (H.K.); matsudak-der@h.u-tokyo.ac.jp (K.M.M.); NORIMATSUY-DER@h.u-tokyo.ac.jp (Y.N.); KUZUMIA-DER@h.u-tokyo.ac.jp (A.K.); fukayamam-der@h.u-tokyo.ac.jp (M.F.); FUKASAWAT-DER@h.u-tokyo.ac.jp (T.F.); EBATAS-DER@h.u-tokyo.ac.jp (S.E.); YOSHIZAKIA-DER@h.u-tokyo.ac.jp (A.Y.-O.); ASANOY-DER@h.u-tokyo.ac.jp (Y.A.); SATOS-DER@h.u-tokyo.ac.jp (S.S.); 2 Department of Biostatistics, School of Public Health, Graduate School of Medicine, The University of Tokyo, Tokyo 113-8655, Japan; oba@epistat.m.u-tokyo.ac.jp

**Keywords:** calponin 3, systemic sclerosis, fibrosis, skin sclerosis, arthralgia

## Abstract

Systemic sclerosis (SSc) is a connective tissue disease characterized by tissue fibrosis and vasculopathy in various organs with a background of inflammation initiated by autoimmune abnormalities. Calponin 3 plays a role in the cell motility and contractibility of fibroblasts during wound healing in the skin. We aimed to evaluate serum calponin 3 levels in SSc patients and their association with clinical manifestations of SSc. Serum samples were collected from 68 patients with SSc and 20 healthy controls. Serum calponin 3 levels were examined using enzyme-linked immunosorbent assay kits, and their association with clinical features of SSc was statistically analyzed. The upper limit of the 95% confidence interval of serum calponin 3 levels in healthy controls was utilized as the cut-off value when dividing SSc patients into the elevated and normal groups. Serum calponin 3 levels were significantly higher in SSc patients than in healthy controls (mean (95% confidence interval), 15.38 (14.66–16.11) vs. 13.56 (12.75–14.38) ng/mL, *p* < 0.05). The modified Rodnan total skin thickness score was significantly higher in the elevated serum calponin 3 level group than in the normal level group (median (25–75th percentiles), 10.0 (2.0–16.0) vs. 6.5 (3.25–8.75), *p* < 0.05). Moreover, SSc patients with increased serum calponin 3 levels also had a higher frequency of arthralgia (40% vs. 9%, *p* < 0.05). Elevated serum calponin 3 levels were associated with skin sclerosis and arthralgia in SSc patients. Serum calponin 3 levels might be a biomarker that reflects the severity of skin sclerosis and joint involvement in SSc.

## 1. Introduction

Systemic sclerosis (SSc) is a connective tissue disease characterized by excessive fibrosis of the skin, lung, esophagus, and other internal organs [1]. Although the pathogenesis of SSc remains unclear, a triad of immunological abnormality, tissue fibrosis, and vascular damage is considered the primary feature of SSc [2,3]. In the time course of SSc, immune cell infiltration and vascular injury occur in the early stage, followed by excessive deposition of extracellular matrix in tissues, resulting in organ fibrosis [4]. SSc also causes various painful symptoms, such as Raynaud’s phenomenon, pitting scars, digital ulcerations, and arthralgia, other than organ fibrosis, which severely compromises the SSc patients’ quality of life [5].

Calponin, a family of actin filament-associated proteins first identified in smooth muscle, has three isoforms: calponin 1, calponin 2, and calponin 3, also known as basic, neutral, and acidic calponin, respectively [6,7,8]. Calponin 1 is well known to be highly specific to smooth muscle cells. Recent studies reported the expression of calponin 1 in non-muscle cells, such as types of mesenchymal cell [9,10]. Interestingly, it has been reported that the expression of calponin 1 is significantly increased in the skin tissue of SSc patients and knockdown of calponin 1 in the dermal fibroblasts inhibits cell proliferation and reduces the protein expression of collagen [11].

Calponin 3 is expressed in smooth muscle cells and various types of non-muscle cell. Calponin 3 is known to play a role in the plasticity of neural tissues [12] and cell fusion of trophoblasts and myoblasts during embryonic development [13,14]. A recent study showed that the expression of calponin 3 is increased and localized in the plasma membrane throughout early B cell development, whereas its physiological function is still unclear [15].

In the skin, calponin 3 is markedly expressed by dermal fibroblasts and myofibroblasts in the proliferation phase of wound healing. Downregulation of calponin 3 in primary fibroblasts impairs stress fiber formation, resulting in a reduction of cell motility and contractile ability [16]. These insights suggest that calponin 3 is involved in the remodeling and homeostasis of cutaneous tissues. However, no association of calponin 3 with skin diseases, including SSc, has yet been elucidated. Here, we report the serum levels of calponin 3 and their clinical correlations in SSc patients, suggesting the possible utility of serum calponin 3 levels as a biomarker that reflects the severity of skin sclerosis.

## 2. Experimental Section

### 2.1. Serum Samples from Systemic Sclerosis (SSc) Patients and Healthy Controls

Serum samples were collected from 68 patients with SSc (6 men and 62 women; median age of 59 years (25–75th percentiles: 48–68 years); median disease duration of 3.0 years (25–75th percentiles: 1–9.75 years)) and 20 healthy controls (3 men and 17 women; median age of 53 years (25–75th percentiles: 43–66 years)) after obtaining written informed consent. All SSc patients fulfilled the American College of Rheumatology and European League Against Rheumatism criteria for the classification of the disease [17]. None of the patients was being treated with oral corticosteroids or immunosuppressants prior to sample collection. SSc patients were categorized by LeRoy’s classification rule [1] into 38 diffuse cutaneous SSc (dcSSc) patients and 30 limited cutaneous SSc (lcSSc) patients. Fresh venous blood samples were centrifuged shortly after clot formation. All samples were stored at −80 °C prior to use. The study was approved by the ethical committee of The University of Tokyo Hospital (No. 0695).

### 2.2. Measurement of Serum Calponin 3 Levels

Serum calponin 3 levels were examined using enzyme-linked immunosorbent assay kits (MyBioSource, San Diego, CA, USA). First, polystyrene plates were coated with anti- calponin 3 antibodies and incubated for one hour at 37 °C with 4-fold diluted serum or calponin 3-Horse Radish Peroxidase (HRP) conjugate to obtain the standard curve. Second, the wells were washed and incubated with HRP enzyme substrate for 20 min at 37 °C. Finally, stop solution was added to terminate the reaction, and the absorbance at 450 nm was measured. Serum calponin 3 levels were calculated from a standard curve.

### 2.3. Clinical Assessments of Patients

The clinical data of SSc patients were gathered by a retrospective review of medical records. Disease duration was defined as the interval between the first clinical event, which is a clear manifestation of SSc other than Raynaud’s phenomenon, and the time serum was obtained. The severity of skin sclerosis was examined by the modified Rodnan total skin thickness score (mRSS). Esophageal dysfunction was defined as findings of reflux esophagitis on gastrointestinal endoscopy or hypomotility on barium-contrast radiography. Interstitial lung disease was defined as bibasilar interstitial fibrosis on chest radiographs or alveolitis on high-resolution computed tomography.

### 2.4. Statistical Analysis

Statistical analysis was performed using the Kruskal–Wallis test for multiple comparison, Mann–Whitney’s U-test for two-group comparisons, and Fisher’s exact probability test for comparison of frequency. Spearman’s rank correlation analysis was used to examine the relationship between two continuous variables. *p* < 0.05 was considered statistically significant.

## 3. Results

### 3.1. Serum Calponin 3 Levels Were Elevated in SSc

**Figure 1:**
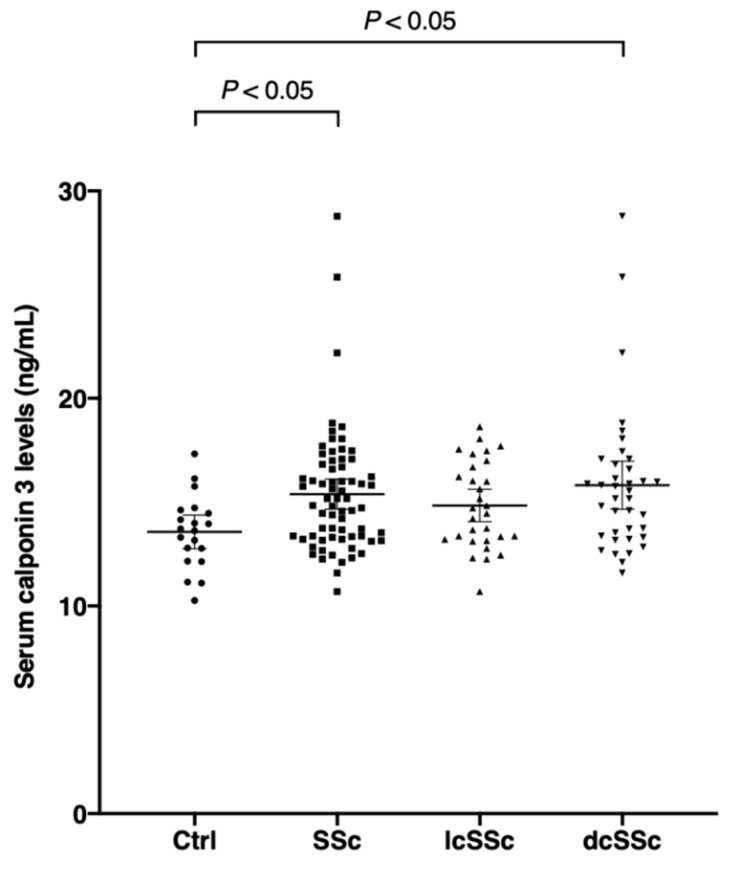
Serum calponin 3 levels in systemic sclerosis (SSc), diffuse cutaneous systemic sclerosis (dcSSc), limited cutaneous systemic sclerosis (lcSSc), and healthy controls (Ctrl). Serum calponin 3 levels were measured by a specific enzyme-linked immunosorbent assay. The horizontal line in each column shows the mean. The Kruskal–Wallis test was conducted for multiple-group comparison.

Serum calponin 3 levels in SSc patients (15.38 ng/mL; 95% confidence interval [CI], 14.66 to 16.11) were significantly higher than those in healthy controls (13.56 ng/mL; 95% CI, 12.75 to 14.38, *p* < 0.05; Figure 1). For the SSc subgroups, serum calponin 3 levels in dcSSc patients (15.81 ng/mL; 95% CI, 14.66 to 16.97) were significantly increased compared to those in healthy controls (13.56 ng/mL; 95% CI, 12.75 to 14.38, *p* < 0.05), while there were no significant differences in the serum calponin 3 levels between lcSSc and healthy controls (*p* = 0.29). Furthermore, there was no significant difference in serum calponin 3 levels between dcSSc and lcSSc.

### 3.2. Clinical Features of SSc Patients with Elevated Serum Calponin 3 Levels

Clinical and laboratory features were compared between SSc patients with elevated serum calponin 3 levels and those with normal levels (Table 1). The cut-off value was set at 14.38 ng/mL (upper limit of the 95% confidence interval of serum calponin 3 levels in healthy controls). There were no significant differences in sex, age, disease duration, and the proportion of dcSSc patients between the two groups of patients with elevated and normal serum calponin 3 levels. On the clinical features, the mRSS with elevated serum calponin 3 levels was significantly higher than in those with normal levels (median (25–75th percentiles), 10.0 (2.0–16.0) vs. 6.5 (3.25–8.75), *p* < 0.05; Supplementary Figure S1). Moreover, patients with elevated serum calponin 3 levels had a higher frequency of arthralgia (40% vs. 8.7%, *p* < 0.05; Supplementary Figure S2). Meanwhile, the frequencies of Raynaud’s phenomenon, pitting scar/ulcers, nail fold bleeding, esophageal dysfunction, and pulmonary hypertension were comparable between the two groups. On the laboratory findings, the presence of anti-topoisomerase I, anti-centromere, or anti-RNA polymerase III antibody did not show significant differences between the two groups. Serum levels of C-reactive protein and erythrocyte sedimentation rate, median tended to be higher in the elevated serum calponin 3 level group than in the normal group, but the difference was not statistically significant.

**Figure 2:**
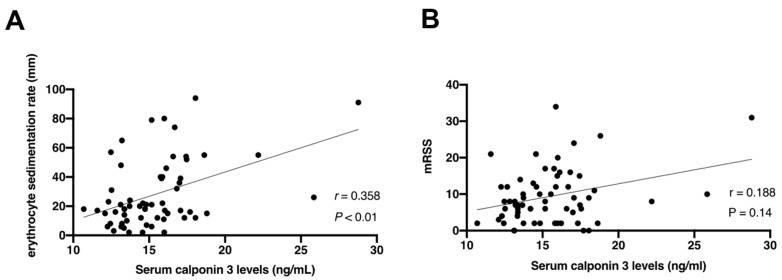
Correlation between serum calponin 3 levels with (**A**) erythrocyte sedimentation rate and (**B**) modified Rodnan total skin thickness score (mRSS) in systemic sclerosis. The solid line shows the regression line. Spearman’s rank correlation coefficient (r) was calculated for correlation analysis.

In addition, we examined the correlation between serum calponin 3 levels and these clinical features and laboratory findings in SSc patients. As a result, a significant correlation with serum calponin 3 levels was observed only in erythrocyte sedimentation rate (*r* = 0.358, *p* < 0.01; Figure 2A), but not in mRSS (*r* = 0.188, *p* = 0.14; Figure 2B).

## 4. Discussion

Our investigation revealed that serum calponin 3 levels were significantly elevated in dcSSc patients compared to those in healthy controls. Moreover, SSc patients with elevated serum calponin 3 levels had higher skin thickness score measured by mRSS, and frequency of arthralgia. These results suggest that calponin 3 may play a role in the development of skin sclerosis and joint involvement in SSc patients.

Previous studies have shown that calponin 3 is upregulated in dermal fibroblasts and myofibroblasts during wound healing and is involved in cell motility and contractibility [16,18]. Fibroblasts are activated by multiple cytokines and growth factors such as transforming growth factor β (TGF-β) to generate myofibroblasts. Dysregulated TGF-β signaling in fibroblasts and myofibroblasts, resulting in the production of extracellular matrices such as collagen, has been observed in multiple studies of SSc patients [19,20]. Intriguingly, calponin 3 is constitutively associated with extracellular signal-regulated kinase (ERK)1/2, which is known as a member of the mitogen-activated protein kinase (MAPK) family and a downstream target of the TGF-β signaling pathway, in which downregulation of calponin 3 leads to the inhibition of ERK1/2 activity in fibroblasts [21]. These observations suggest that elevated calponin 3 levels in SSc might be a positive regulator of the fibroblast homeostasis that leads to the development of skin sclerosis.

Joint involvement is a major symptom in SSc patients. Joint symptoms have been noted in 12–66% of patients at the time of diagnosis, and they most frequently manifest as arthralgia [22,23,24,25]. However, in SSc, arthralgia does not usually show any joint modifications on conventional X-rays, although mild synovitis can be detected on magnetic resonance imaging [26]. Synovial biopsies from SSc joints showed histologically inflammatory cell infiltration, while severe fibrosis of synovial tissue was observed in the late phase of the disease [27]. A recent study demonstrated that calponin 3 negatively regulates bone morphogenetic protein (BMP) signaling by interacting with the Smad proteins 1 and 5 in cartilage tissue [28]. BMP signaling plays a critical role in both chondrogenesis and osteogenesis [29]. Therefore, calponin 3 may contribute to cartilage tissue homeostasis by regulating BMP signaling. Our study showed that serum calponin 3 levels were positively associated with arthralgia frequency. Serum calponin 3 levels may be a useful biomarker as an objective measure that reflects the severity of joint involvement, including arthralgia.

This study has several potential limitations. First, the total sample size was relatively small and, in particular, there were fewer male than female samples in SSc, which substantially restricted the range of behaviors that could be studied statistically. Second, the serum calponin 3 levels of SSc patients were only measured once before the administration of oral corticosteroids or other immunosuppressants. Further longitudinal studies are needed to determine whether serum calponin 3 levels are altered after treatment. Third, our study did not address the assessment of joint involvement in detail, which would require future studies to clarify the correlation of serum calponin 3 levels with the prevalence of each type of joint involvement such as arthritis. Finally, this study did not reveal the expression and localization of calponin 3 by immunohistochemical staining of skin biopsies. Interestingly, a recent study showed that expression of calponin 1, which is a member of the calponin family is increased in the skin tissue of SSc patients [11]. Further studies will be needed to clarify the localization and pathological role of the calponin family, including calponin 1 and calponin 2 as well as calponin 3 in the development of SSc.

In summary, this is the first study to indicate that serum calponin 3 levels were elevated in SSc. Interestingly, a recent study showed that calponin 3 was identified as a novel autoantigen, and anti-calponin 3 antibodies were detected in patients with several autoimmune diseases, such as Sjögren’s syndrome, systemic lupus erythematosus, myositis, and multiple sclerosis [30]. Therefore, calponin 3 has the potential to be an autoantigen of not only these autoimmune diseases but also SSc. Although further studies are required to clarify the role of calponin 3 in the pathogenesis of SSc, our results suggest that serum calponin 3 levels might be a valuable biomarker that reflects the severity of skin sclerosis and joint involvement in SSc.

## Figures and Tables

**Table 1:** Clinical features of SSc patients stratified by serum calponin 3 levels.

Characteristic	Elevated Calponin 3 (*n* = 41)	Normal Calponin 3 (*n* = 27)	*p*
Sex, male/female	4/37	2/25	NS
Age, median (IQR) years	60 (49–70)	58 (47–66)	NS
Disease duration, median (IQR) years	3.0 (1–7)	3.2 (1.5–10)	NS
No. with dcSSc/no. with lcSSc	25/16	13/14	NS
**Clinical features**			
mRSS, median (IQR)	10.0 (2.0–16.0)	6.5 (3.25–8.75)	0.04
Raynaud’s phenomenon, %	90	93	NS
Nail fold bleeding, %	80	80	NS
Pitting scar/ulcers, %	35	41	NS
Interstitial lung disease, %	51	44	NS
esophageal dysfunction, %	73	81	NS
Pulmonary hypertention, %	2.4	0	NS
Arthralgia, %	40	8.7	0.01
**Laboratoly findings**			
Anti-topoisomerase I, %	46	52	NS
Anti-centromere, %	37	37	NS
Anti-RNA polymerase III, %	12	7	NS
C-reactive protein, median (IQR) mg/dL	0.08 (0.04–0.30)	0.04 (0.01–0.04)	NS
Erythrocyte sedimentation rate, median (IQR) mm	22.0 (12.0–52.0)	17.5 (8.0–23.75)	NS

N: number, NS: not significant, IQR: interquartile range, SSc: systemic sclerosis, dcSSc: diffuse cutaneous SSc, lcSSc: limited cutaneous SSc, mRSS: modified Rodnan total skin thickness score. Statistical analysis was carried out by Mann–Whitney’s U-test for continuous variables and Fisher’s exact probability test for comparison of frequency.

## Data Availability

The data presented in this study are available on request from the corresponding author. The data are not publicly available due to ethical reason.

## Supplementary Materials

The following are available online at https://www.mdpi.com/2077-0383/10/2/280/s1, Figure S1: Graphical representation (box plot) of modified Rodnan total skin thickness score (mRSS) in systemic sclerosis(SSc) patients with elevated serum calponin 3 levels and those with normal levels. Statistical analysis was carried out by Mann-Whitney’s U-test for the two-group comparisons., Figure S2: The number of patients with or without arthralgia in systemic sclerosis(SSc) patients with elevated serum calponin 3 levels and those with normal levels. Statistical analysis was carried out by Fisher’s exact probability test for comparison of arthralgia frequency.
