# Supplementary material for: Serum Calponin 3 Levels in Patients with Systemic Sclerosis: Possible Association with Skin Sclerosis and Arthralgia
Source: J Clin Med. 2021 Jan 14;10(2):280. doi: 10.3390/jcm10020280 (PMC7828654; doi:10.3390/jcm10020280)
Supplement: Supplementary file 1 [file jcm-10-00280-s001.pdf]

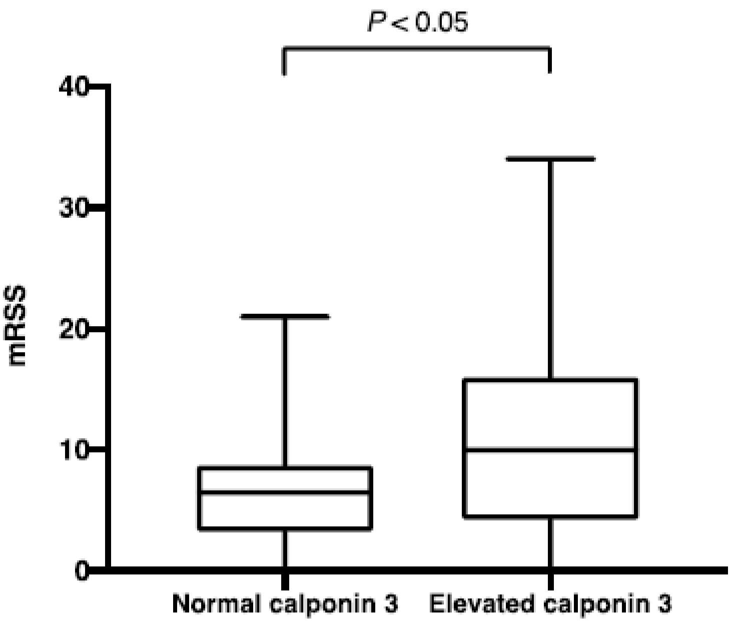

Figure S1. Graphical representation (box plot) of modified Rodnan total skin thickness score (mRSS) in systemic sclerosis(SSc) patients with elevated serum calponin 3 levels and those with normal levels. Statistical analysis was carried out by Mann-Whitney's U-test for the two-group comparisons.

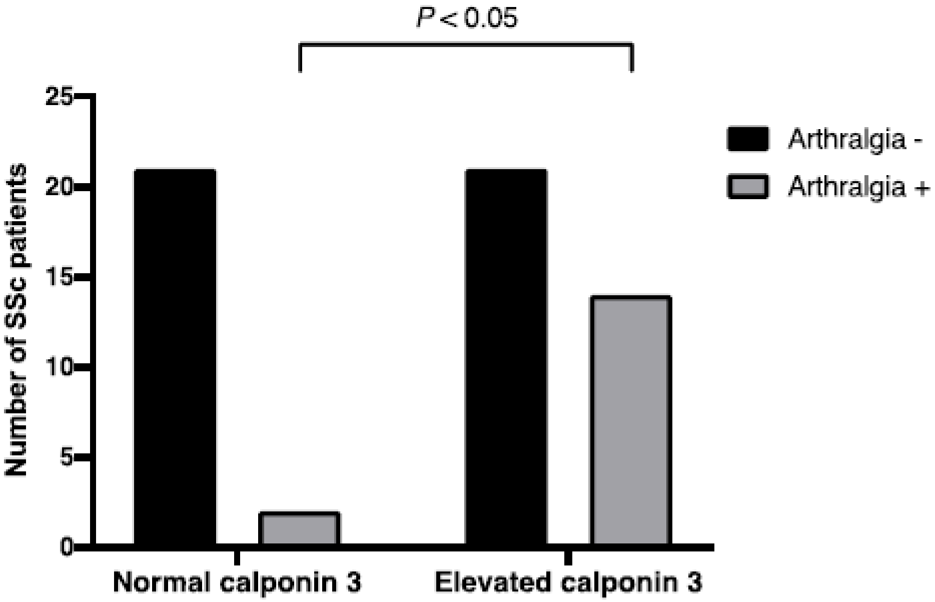

Figure S2. The number of patients with or without arthralgia in systemic sclerosis(SSc) patients with elevated serum calponin 3 levels and those with normal levels. Statistical analysis was carried out by Fisher's exact probability test for comparison of arthralgia frequency.
